# IL-6R/STAT3/miR-204 feedback loop contributes to cisplatin resistance of epithelial ovarian cancer cells

**DOI:** 10.18632/oncotarget.16610

**Published:** 2017-03-27

**Authors:** Xiaolan Zhu, Huiling Shen, Xinming Yin, Lulu Long, Xiaofang Chen, Fan Feng, Yueqin Liu, Peiqing Zhao, Yue Xu, Mei Li, Wenlin Xu, Yuefeng Li

**Affiliations:** ^1^ Department of Gynecologic Oncology, The Fourth Affiliated Hospital of Jiangsu University, Zhenjiang, Jiangsu 212001, China; ^2^ Jiangsu University, Medical School, Zhenjiang, Jiangsu 212003, China; ^3^ Department of Oncology, The Affiliated People Hospital of Jiangsu University, Zhenjiang, Jiangsu 212001, China; ^4^ Department of Radiology, The Affiliated Hospital of Jiangsu University, Zhenjiang, Jiangsu 212001, China

**Keywords:** miR-204, IL-6R, STAT3, chemoresistance, EOC

## Abstract

Enhanced chemoresistance is, among other factors, believed to be responsible for treatment failure and tumor relapse in patients with epithelial ovarian cancer (EOC). Here, we exposed EOC cells to interleukin-6 (IL-6) to activate oncogenic STAT3, which directly repressed miR-204 via a conserved STAT3-binding site near the TRPM3 promoter region upstream of miR-204. Repression of miR-204 was required for IL-6-induced cisplatin (cDDP) resistance. Furthermore, we identified the IL-6 receptor (IL-6R), which mediates IL-6-dependent STAT3 activation, as a direct miR-204 target. Importantly, the resulting IL-6R/STAT3/miR-204 feedback loop was identified in patients with EOC, and its activity correlated with chemosensitivity. Moreover, exogenous miR-204 blocked this circuit and enhanced cDDP sensitivity both *in vitro* and *in vivo* by inactivating IL-6R/STAT3 signaling and subsequently decreasing the expression of anti-apoptotic proteins. Our findings illustrate the function of this feedback loop in cDDP-based therapy and may offer a broadly useful approach to improve EOC therapy.

## INTRODUCTION

Patients with epithelial ovarian cancer (EOC) typically present with advanced disease often relapse, even with optimal debulking and response to adjuvant chemotherapy. However, the mechanisms underlying the chemoresistance and relapse of EOC are poorly understood [1]. Nevertheless, a growing body of evidence suggests that inflammation, promoted chemoresistance [2, 3]. Inflammation was a biological process that was initiated upon injury to remove harmful stimuli and to initiate a healing process [4] However, prolonged inflammation may be harmful to the organism and promote chronic disease, including cancer. For example, approximately 25% of cancers are due to chronic infection or other types of chronic inflammation [5]. Currently, solid tumors, even those arising in the absence of chronic inflammation, are accepted to contain an inflammatory microenvironment [6]. The role for inflammation in ovarian carcinogenesis was first proposed in the ‘incessant ovulation theory’ [7]. Specifically, the rupture of the ovarian surface epithelium induces an inflammatory reaction that leads to cell damage and proliferation, enhances the potential for aberrant DNA repair, inactivates tumor-suppressor genes, and results in subsequent mutagenesis [8]. Interleukin-6 (IL-6), a pro-inflammatory cytokine produced by several types of immune cells and carcinomas, is an important mediator of the tumor-promoting effects of inflammation, especially in later states of tumorigenesis [9, 10]. In fact, high IL-6 levels in ascites during primary surgery predict a high risk for rapid disease relapse and correlate with residual disease after debulking in patients with advanced EOC. In addition to its local effects in the tumor microenvironment, tumor-derived IL-6 can stimulate paraneoplastic thrombocytosis in patients with advanced EOC, which is also associated with poor prognosis [11, 12]. These findings indicate the therapeutic potential of targeting IL-6 and its downstream signaling.

IL-6 acts in both autocrine and paracrine manners by binding to the IL-6 receptor (IL-6R), which consists of the ligand-binding subunit glycoprotein (gp) 80 and the signal-transducing subunit gp130 [13]. This binding leads to the recruitment of the signal transducing subunit gp130 and subsequently activates signal transducer and activator of transcription 3 (STAT3) [14, 15], which is the most common member of the STAT protein family and consistently constitutively activated in cancers. Inflammatory cytokine-mediated STAT3 activation induces STAT3 tyrosine-705 phosphorylation and cytoplasmic-to-nuclear shuttling, the recognition of STAT3-specific DNA binding elements, and the transcriptional activation of target genes [16]. By modulating the transcription of many genes that regulate a wide variety of biological processes, including cell proliferation, apoptosis, metastasis, and the immune response [17], the STAT3 oncoprotein drives tumor progression and chemoresistance in EOC [10]. Importantly, the inhibition of constitutively activated STAT3 induces cell apoptosis, identifying it as an effective target to combat cancer drug resistance [18, 19].

Identification of additional independent biomarkers associated with EOC survival and their functions may offer greater insight into the underlying biology of this disease and the mechanisms of response to treatment and relapse. MicroRNAs (miRNAs), non-coding RNA molecules that regulate gene expression at the post-transcriptional level via sequence-specific base pairing with the 3′UTRs of target mRNA, several of them have been linked to the development of drug resistance in EOC [20, 21]. For example, recent studies have demonstrated that miRNAs can modulate the IL-6/STAT3 signaling pathway, and STAT3 also regulates the expression of miRNAs [18]. One candidate miRNA of interest, miR-204, is located at the cancer-associated genomic region 9q21.1-q22.3 locus and known to be significantly dysregulated in many tumor types, including breast cancer, prostate cancer, kidney cancer, gastric cancer, colorectal cancer, neuroblastoma, glioma, endometrial cancer, and intrahepatic cholangiocarcinoma [22–26], which implicates miR-204 as a tumor suppressor gene. To date, a cohort of genes related to different cancer pathways have been identified as its target genes. Accordingly, miR-204 suppresses tumor cell growth, apoptosis and survival by targeting Mcl-1, Bcl-2 and TrkB [25, 27], impacted tumor migration, invasion and metastasis by targeting FOXC1 and slug [26, 28]. Furthermore, it also reversed stem cell-like phenotype via SOX4 and EphB2 [29]. Additionally, miR-204 was also a VHL-regulated tumor suppressor by inhibiting macro-autophagy via the direct functional targeting of MAP1LC3B [30].

miR-204 and its host gene TRPM3 reportedly share the same regulatory motif for transcription and derive from a single transcription unit [23], and a promoter region analysis of TRPM3 identified three putative STAT-binding sites located nearby the promoter region of TRPM3. Phospho-STAT3 (p-STAT3) was reported to bind to putative STAT-binding sites located near the TRPM3 promoter region upstream of miR-204, which ultimately reduces the levels of miR-204 [25, 31]. Interestingly, bioinformatics software predicted IL-6R to be potential target of miR-204. Thus, IL-6-mediated STAT3 activation may downregulate miR-204, which may reversibly inhibit IL-6R expression to further repress the IL-6R/STAT3 pathway and form a regulatory loop between miRNAs and the IL-6/STAT3 signaling pathway. However, the molecular mechanisms underlying these associations and interactions in EOC chemoresistance warrant further investigation.

In this study, we investigated the contribution of IL-6 stimulation to cDDP resistance in EOC cells. We identified a feedback circuit that is established by the IL-6R/STAT3-mediated repression of miR-204 and upregulation of IL-6R, which enhance IL-6R/STAT3 signaling and promote cDDP resistance in EOC.

## RESULTS

### IL-6 induces cDDP resistance in EOC cells

**Figure 1:**
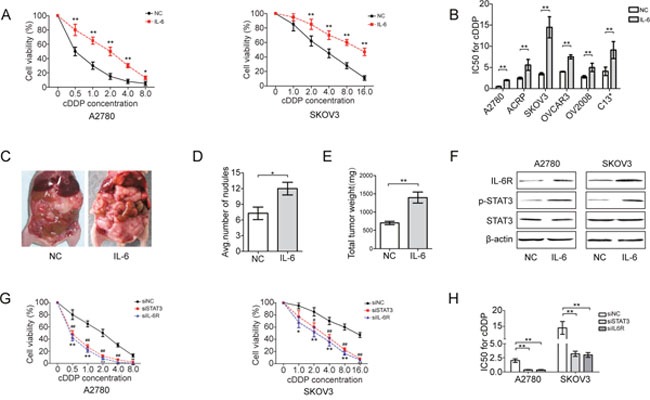
IL-6 induces cDDP resistance of EOC cells **(A)** Viability of EOC cells pretreated with IL-6 (20 ng/ml) for 72 h in response to an increasing concentration of cDDP. **(B)** IC50 for cDDP in EOC cells treated with IL-6 (20 ng/ml). A2780 cells were intraperitoneally injected into BALB/c nude mice (n=6), which were then treated with recombinant IL-6 for 5 weeks. **(C)** Representative images showing the formation of tumors in mice. **(D)** Tumor incidence in indicated mice. **(E)** Total tumor weight per mouse. **(F)** Western blotting analysis of IL-6R, p-STAT3 and STAT3 in EOC cell lines after IL-6 treatment. **(G)** Cell viability of EOC cells transfected with siIL-6R or siSTAT3 following with IL-6 stimulation. **(H)** IC50 for cDDP in EOC cells after transfection. *P< 0.05, **P< 0.01.

The cDDP-resistant EOC cell lines SKOV3, ACRP, OVCAR3, OV2008 and C13* expressed higher levels of IL-6 and IL-6R than the cDDP-sensitive cell line A2780 (Supplementary Figure 1A and 1B). ADM-resistant breast cancer cell lines also expressed more IL-6 and IL-6R than Adriamycin (ADM)-sensitive cell lines (Supplementary Figure 1C). To elucidate the role and function of IL-6 in the cDDP resistance of EOC cells, we modulated its expression in EOC cells. IL-6 treatment was sufficient to induce cDDP resistance in EOC cells, as evidenced by enhanced cell viability (Figure 1A) and an increased IC50 for cDDP (Figure 1B; and Supplementary Figure 1D-1G). *In vivo* treatment with IL-6 also promoted tumor formation and increased tumor nodules in mice (Figure 1C-1E). As expected, recombinant IL-6 phosphorylated STAT3, an effector of IL-6 signaling, presumably activating STAT3 in EOC cells (Figure 1F), which is typical for IL-6-mediated STAT3 activation. Indeed, the RNA interference-mediated downregulation of STAT3 or IL-6R prevented IL-6-induced cDDP resistance (Supplementary Figure 1H; and Figure 1G and 1H), and the transient stimulation of IL-6 strikingly increased IL-6R mRNA expression (Supplementary Figure 1I), suggesting that elevated IL-6 levels may initiate a feedback loop that continuously enhances IL-6R expression and induces a stably transformed phenotype. Overall, these data imply that IL-6 treatment may induce cDDP resistance in EOC cells via a feedback regulatory mechanism.

### IL-6-induced cDDP resistance of EOC cells are mediated by direct repression of miR-204 by STAT3

**Figure 2:**
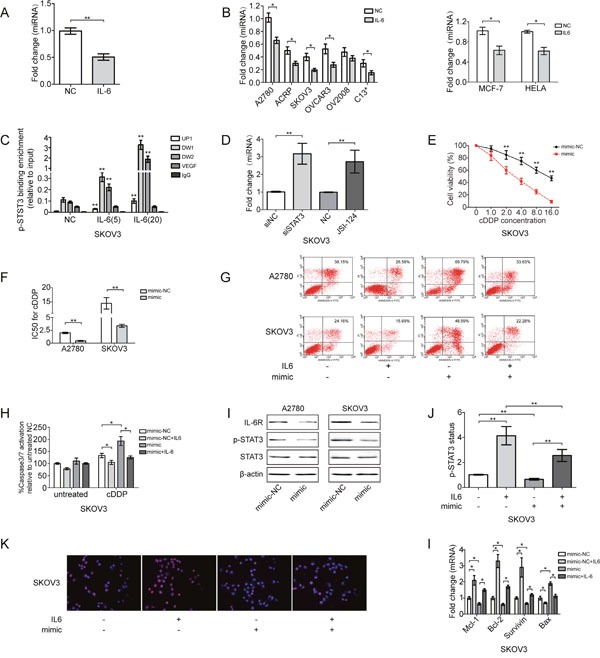
IL-6 induces cDDP resistance of EOC cells through direct repression of miR-204 by STAT3 **(A)** Fold change of miR-204 expression in tumors derived from IL-6-treated mice. **(B)** Fold change of miR-204 in IL-6-treated EOC, HELA and MCF-7 cells. **(C)** ChIP analysis of STAT3 occupancy (fold enrichment) at the TRPM3/miR-204 locus in EOC cells treated with vehicle or IL-6. **(D)** qRT-PCR analysis of miR-204 in EOC cells treated with siSTAT3 or JSI-124 (5 μg/ml) for 24 h and subsequently treated with IL-6 for 72 h. EOC cells were treated with mimic for 24 h and subsequently treated with IL-6 for 72 h; cell viability **(E)**, changes in the IC50 of cDDP **(F)**, cell apoptosis **(G)**, and caspase 3/7 activation **(H)** were measured in EOC cells. **(I)** Western blotting analysis of IL-6R, p-STAT3 and STAT3 in EOC cell lines. **(J)** STAT3 phosphorylation status (Tyr 705) was assessed by ELISA in SKOV3 cells. **(K)** miR-204 suppressed IL-6-promoted p-STAT3 nuclear translocation, as determined by immunofluorescence (200x magnification). p-STAT3 (red), nucleus/DAPI (blue), co-localization (pink). **(l)** qRT-PCR analysis of indicated mRNAs in SKOV3 cells. *P< 0.05, **P< 0.01.

To determine whether IL-6-induced cDDP resistance of EOC cells involved in repression of miR-204, we analyzed miR-204 expression in EOC cells after IL-6 treatment *in vivo* and *in vitro*. Indeed, miR-204 expression decreased in tumor tissue obtained from IL-6-treated mice and in EOC cells exposed to IL-6 (Figure 2A and 2B). Exposing HeLa cervical cancer cells and MCF-7 breast cancer cells to IL-6 also repressed miR-204 expression (Figure 2B). Therefore, this effect was not restricted to EOC cells but is presumably a general response of cancer cells. We also tested the ability of p-STAT3 to bind to the reported three putative STAT-binding sites located near the TRPM3 promoter region upstream of miR-204 in EOC cells [25, 31]. As expected, ChIP assays showed that p-STAT3 directly bound to all three putative STAT-binding sites in A2780 and SKOV3 cells, and binding to DW1 was most pronounced. Moreover, increasing IL-6 stimulation significantly increased STAT3 occupancy at the miR-204 promoter (Figure 2C; and Supplementary Figure 2A and 2B). IL-6-induced STAT3 activation strongly reduced miR-204 expression levels, which was prevented by the pharmacological inhibition of STAT3 (JSI-124) or the siRNA-mediated downregulation of STAT3, demonstrating that STAT3 mediates the repression of miR-204 after IL-6 exposure (Figure 2D; and Supplementary Figure 2C).

This intriguing feature of miR-204 pushed us to further investigate its role in IL-6-induced cDDP resistance. Specifically, the ectopic expression of miR-204 prevented the IL-6-induced cDDP resistance of EOC cells, as indicated by reduced cell viability, increased cell apoptosis and increased caspase3/7 activation (Figure 2E-2H; and Supplementary Figure 2D-2F). miR-204 also blocked IL-6-induced STAT3 activation, as measured by reduced STAT3 phosphorylation levels (Figure 2I and 2J; and Supplementary Figure 2G). We also evaluated the level of p-STAT3 that translocated to the nucleus, which is associated with its transcriptional activity, by immunofluorescence. IL-6 increased the number of p-STAT3-positive cells (presenting increased nuclear p-STAT3 staining intensity compared with that of control cells), and miR-204 treatment a significantly decreased p-STAT3 nuclear translocation (Figure 2K), suggesting that miR-204 inhibits the transcriptional activity of this protein. In addition, IL-6 treatment also promoted the rearrangement of anti-apoptotic genes (Mcl-1, Bcl-2 and survivin) and reduced the expression of the pro-apoptotic gene Bax, and these effects was reversed by miR-204 expression (Figure 2L). In summary, the repression of miR-204 by STAT3 is required for IL-6-mediated cDDP resistance in EOC cells. Moreover, IL-6 induced IL-6R expression was prevented by ectopic miR-204 (Supplementary Figure 2H), demonstrating that it required and was presumably mediated by repression of miR-204.

### IL-6R is a direct regulator of miR-204

**Figure 3:**
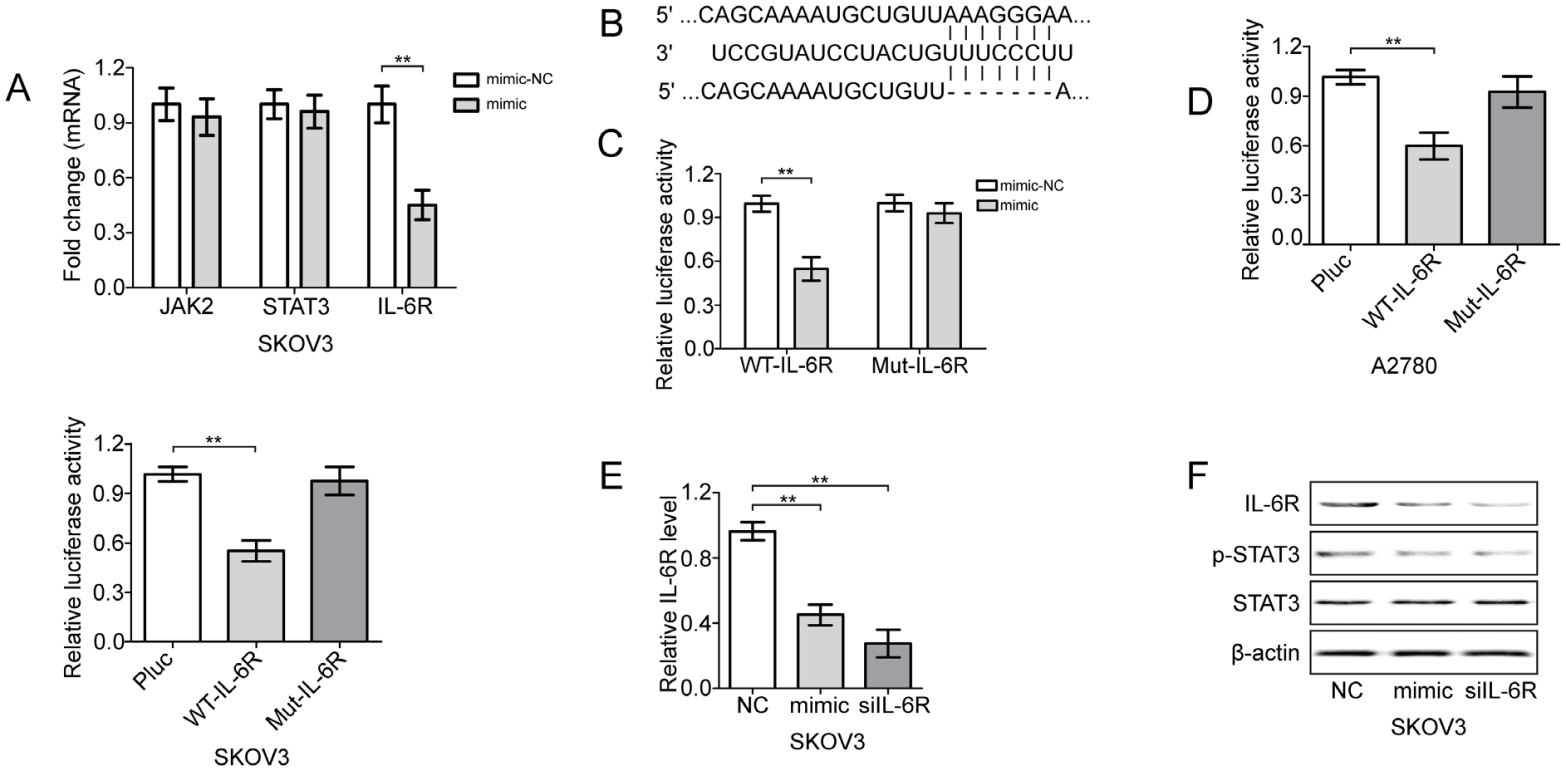
miR-204 targets IL-6R in EOC cells **(A)** Fold change in the indicated mRNAs in mimic-treated SKOV3 cells. **(B)** Specific locations of miR-204 binding sites in the IL-6R 3′UTR (upper and middle sequence) and an ideograph of the mutated IL-6R 3′-UTR with 7 seed nucleotide deletions are shown (bottom sequence). Luciferase activity of the wild-type (WT) or mutant (Mut) IL-6R 3′UTR reporter gene in HEK293 **(C)**, A2780 and SKOV3 cells **(D)** with ectopic miR-204 expression. Evaluation of IL6R mRNA **(E)** and protein **(F)** levels in SKOV3 cells treated with mimic or siIL-6R. **P< 0.01.

Because miR-204 suppressed STAT3 activation, we hypothesized that miR-204 targets one of the components of the IL-6/STAT3 pathway. In support of this hypothesis, we expressed miR-204 in SKOV3 cells to detect changes in IL-6R, JAK2 and STAT3 mRNA expression. The ectopic expression of miR-204 resulted in the repression of IL-6R, but not JAK2 or STAT3 mRNA (Figure 3A). In addition, a sequence complementarity and conservation analysis also revealed that IL-6R may be a potential direct gene target of miR-204 (Figure 3B). Accordingly, the co-transfection of the luciferase IL-6R 3′ UTR reporter construct with miR-204 significantly repressed the activity of the reporter construct containing the wild-type, but not the mutated, miR-204 binding site (Figure 3C and 3D). Furthermore, the significant inverse correlation between the steady-state levels of IL-6R protein and miR-204 in EOC cells also supported these findings (Supplementary Figure 3A and 3B). In addition, the overexpression of miR-204 significantly reduced the IL-6R mRNA and protein levels, and similar effects were identified when IL-6R was suppressed (Figure 3E and 3F), whereas miR-204 inhibition resulted in converse effects on IL-6R expression (Supplementary Figure 3C and 3D). Taken together, we showed that IL-6R mRNA expression was directly regulated by miR-204 via conserved seed-matching sequences. These results indicate the existence of a feedback loop activated by the IL-6-mediated repression of miR-204 and subsequent activation of IL-6R and STAT3, which maintain the repression of miR-204 (Supplementary Figure 3E).

### miR-204 sensitizes EOC cells to cDDP by modulating the IL-6R/STAT3 pathway

**Figure 4:**
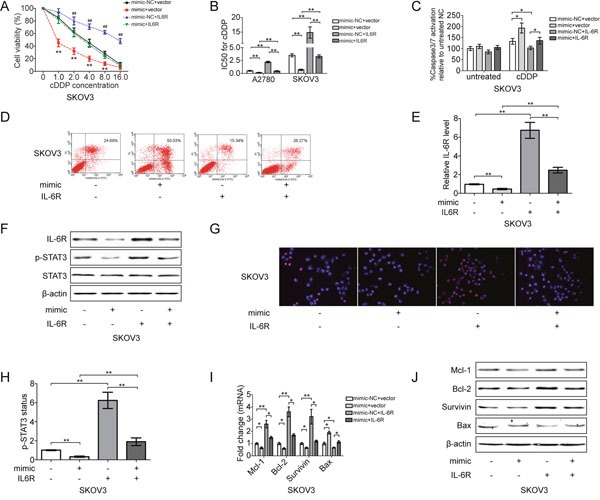
miR-204 sensitizes EOC cells to cDDP by inhibiting IL-6R/STAT3 signaling SKOV3 cells were transfected with mimic-NC and empty vector (NC+vector), mimic-NC and IL-6R recombinant plasmid (NC+IL-6R), mimic and empty vector (mimic+vector), or mimic and IL-6R recombinant plasmid (mimic+IL-6R). After transfection, changes in the cell viability **(A)**, IC50 of cDDP **(B)**, caspase 3/7 activation **(C)** and cell apoptosis **(D)** were analyzed. **(E)** Changes in IL-6R mRNA expression. **(F)** The protein levels of IL-6R, p-STAT3 and STAT3, as detected by Western blotting. **(G)** p-STAT3 nuclear translocation was analyzed by immunofluorescence (200x magnification). p-STAT3 (red), nucleus/DAPI (blue), co-localization (pink). **(H)** STAT3 phosphorylation status (Tyr 705) evaluated by ELISA. Analysis of indicated mRNAs **(I)** and protein **(J)** levels in SKOV3 cells. *P<0.05, **P< 0.01.

To examine the dynamics of this circuit during the cDDP sensitivity of EOC cells, SKOV3 cells were transiently transfected with miR-204. The re-expression of miR-204-induced cell apoptosis, reduced cell viability and enhanced caspase3/7 activation, whereas this effect was partially or completely reversed by IL-6R overexpression (Figure 4A-4D; and Supplementary Figure 4A-4D). Ectopic miR-204 also inhibited IL-6R expression and the phosphorylation and nuclear translocation of STAT3, a downstream target of IL-6R, and this inhibition was rescued by IL-6R expression (Figure 4E–4H; and Supplementary Figure 4E and 4F). In addition to IL-6R, we found that miR-204 expression reduced the expression of anti-apoptotic genes (Mcl-1, Bcl-2 and survivin) and upregulated the expression of pro-apoptotic gene Bax (Figure 4I and 4J), suggesting that miR-204 regulates STAT3 activity by affecting IL-6R expression.

### miR-204 sensitizes EOC cells to cDDP *in vivo*

**Figure 5:**
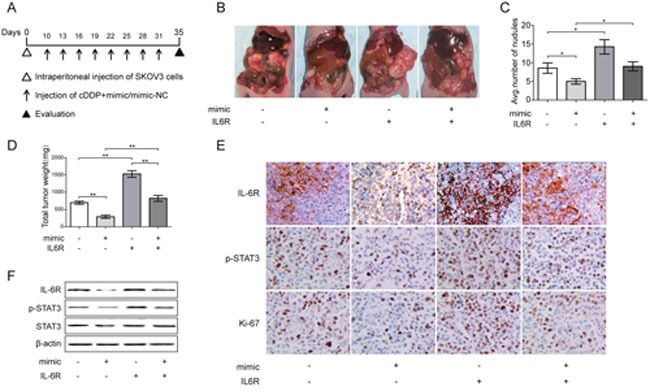
Effect of miR-204 on tumor growth *in vivo* combined with cDDP **(A)** The experimental schedule for the miR-204 and cDDP combination treatment. **(B)** The gross morphology of tumors in nude mice on day 35 after SKOV3 cells injection. **(C)** Tumor incidence in indicated mice. **(D)** Total tumor weight per mouse. **(E)** Immunohistochemistry analyses for IL-6R, p-STAT3, and Ki67 staining were performed on SKOV3 xenograft tumor sections. Representative staining is shown (200x magnification). **(F)** Representative images of the protein expression of IL-6R, p-STAT3, and STAT3 in tumors collected from each group, as detected by Western blotting. *P< 0.05, **P< 0.01.

Next, the therapeutic effect of miR-204 was investigated *in vivo*. Briefly, BALB/c nude mice were intraperitoneally injected with transfected SKOV3 cells, and we allowed potential tumors to grow for one week before intraperitoneally administering cDDP therapy twice per week for 4 consecutive weeks (Figure 5A). The mice receiving combined treatment with cDDP and mimic showed an overall reduction in tumor burden compared with mice in the NC group (Figure 5B). Moreover, the total numbers of nodules on the surfaces of organs, including the stomach, liver, spleen, intestines, diaphragm, omentum and abdominal wall, were markedly reduced in the mimic group (Figure 5C and 5D), whereas the upregulation of IL-6R promoted tumor formation in mice and increased the number of tumor nodules. In addition, the enforced expression of miR-204 together with downregulated expression of p-STAT3, IL-6R and Ki-67 was confirmed in mimic treated tumors (Figure 5E-5F; and Supplementary Figure 5A-5B). Taken together, these data indicate that miR-204 acquisition inactivated IL-6R/STAT3 signaling in EOC and improved the efficacy of cDDP therapeutic effects. In combination with the functional analysis of human EOC cells described above, these findings strengthen the notion that concurrent modulation of miR-204 target genes may be a therapeutically effective adjunctive treatment with chemotherapy for EOC tumors and may benefit patients who harbor miR-204-downregulated EOC tumors.

### miR-204 correlates with improved prognosis through IL-6R targeting in EOC

**Figure 6:**
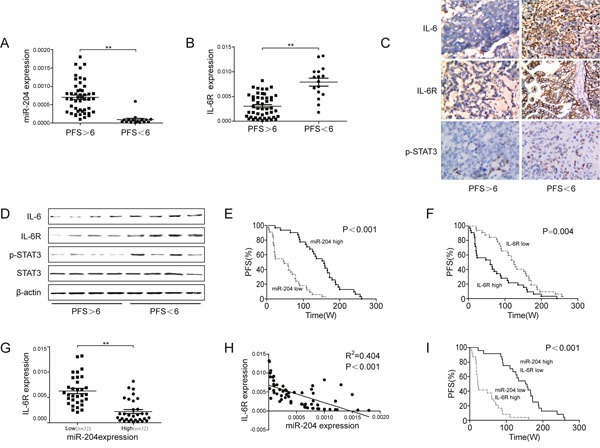
Prognostic role of miR-204 in ovarian cancer patients Relative miR-204 **(A)** or IL-6R **(B)** levels in patient tumor specimens with progression-free survival (PFS) <6 months versus PFS >6 months. **(C)** Immunohistochemistry analysis of IL-6, IL-6R, and p-STAT3 in ovarian cancer specimens obtained from cDDP-sensitive (PFS<6) and cDDP-resistant (PFS>6) patients. Representative images are shown (200x magnification). **(D)** Western blotting analysis of IL-6, IL-6R, p-STAT3, and STAT3 in patient tumor specimens with PFS<6 months (tissues from 4 patients mixed into 1 sample, n=16) versus PFS>6 months (tissues from 12 patients mixed into 1 sample, n=48). A Kaplan-Meier analysis of the PFS of patients with ovarian cancer with the corresponding expression profiles of IL-6R **(E)** and miR-204 **(F)** is shown. **(G)** IL-6R and miR-204 expression were inversely correlated in ovarian cancer samples (n=64). **(H)** A plot of the relative expression of miR-204 vs. IL-6R showed an inverse correlation between IL-6R and miR-204 expression. The correlation index, R^2^, was calculated using the Spearman rank test (R^2^=0.404, *P*<0.001). **(I)** A Kaplan-Meier analysis for the PFS of ovarian cancer patients according to anticorrelation miR-204 and IL-6R expression. **P< 0.01.

To test whether the mechanisms described above for EOC cell lines were also clinically relevant, we examined primary EOC specimens derived from 64 EOC patients. cDDP-resistant patients (PFS<6) exhibited higher IL-6R expression and lower miR-204 expression (Figure 6A and 6B). The IL-6, IL-6R, p-STAT3 protein levels were also elevated in cDDP-resistant patients (Figure 6C and 6D). Similar to high miR-204 expression, low IL-6 and IL-6R expression was associated with longer PFS (Figure 6E-6F, Supplementary Figure 6A). Using the median expression value of miR-204 as a cutoff point, the cohort was dichotomized into miR-204-high- or miR-204-low-expressing tumors. IL-6R expression were directly and significantly anticorrelated with miR-204 levels (Figure 6G), as indicated by Spearman's correlation analysis, an inverse correlation (R^2^=0.404, P<0.001) was observed between miR-204 and IL-6R (Figure 6H), suggesting miR-204-dependent regulation of IL-6R. Then, the 64 patients were stratified into four equal groups based on the expression levels of miR-204 and IL-6R (each group contains 25% of the patients) for further statistical analysis. High miR-204 expression and concurrent low IL-6R expression was significantly associated with a longer PFS in comparison with low miR-204 expression and high IL-6R expression (Figure 6I). Furthermore, we initial surgery before chemotherapy with Taxol and cDDP (1^st^ surgery) with those at the time of recurrence (2^nd^ surgery) in 10 patients with EOC for whom surgical material. In 9 of the 10 samples, the expression of miR-204 was reduced, whereas it was increased in the remaining sample (Supplementary Figure 6B). Moreover, IL-6R expression after chemotherapy at the time of recurrence was increased in 8 of the 10 samples (Supplementary Figure 6C). Furthermore, the clinicopathological features of these two groups did not significantly differ (Supplementary Table 1). Taken together, these data support the hypothesis that the miR-204-dependent regulation of IL-6R/STST3 is associated with cDDP sensitivity and the prognosis of EOC patients.

## DISCUSSION

Despite impressive initial clinical responses, the majority of EOC patients eventually develop some degree of resistance to cDDP-based therapy, which results in a high relapse rate. Moreover, relapsed disease is usually refractory to further treatment, and the mechanisms underlying relapse are not fully understood [32]. Consequently, finding molecular markers that can predict the benefits of chemotherapy and establish a novel strategy to overcome this resistance in patients with EOC patients may reduce discomfort, toxicity and treatment costs.

Most chemotherapeutic agents, including cDDP, 5-FU, and ADM, ultimately kill tumor cells by inducing apoptosis, irrespective of distinct antitumor mechanisms [33]. However, chronic inflammatory stimuli and increased STAT3 activation have been shown to block cDDP-induced apoptosis, which impacts the course of ovarian tumor development and maintains the chemoresistance phenotype of EOC cells [34, 35]. Accordingly, high IL-6 levels in ascites predicted a shorter PFS in patients with EOC, and the therapeutic potential of targeting IL-6 and downstream signaling has been shown in mouse models of ovarian cancer [11, 36, 37]. In addition, tumor-derived products can activate STAT3 signaling in myeloid cells, which may inhibit DC maturation and the stimulation of MDSC development as well as promote tumor growth [38, 39].

Several miRNAs have been demonstrated to modulate IL-6-mediated cell survival and proliferation. Among them, let-7a was confirmed to contribute to the pro-survival effects of enforced IL-6 activity in malignant cholangiocytes [40]. Moreover, miR-26a suppresses the IL-6-induced growth and metastasis of hepatocellular carcinoma by inhibiting STAT3 [41]. In this work, our data revealed the dynamics of a complex molecular self-reinforcing circuit that involved IL-6, IL-6R, STAT3 and miR-204. Specifically, we identified that cells with a cDDP-resistance phenotype produced high levels of IL-6, and an IL-6-triggered feedback loop that involved the STAT3-mediated repression of miR-204 controlled chemoresistance in EOC cells. IL-6 stimulated STAT3 activation to promoted a pro-proliferative and anti-apoptotic program, which inhibited cDDP-induced cytotoxicity by downregulating miR-204 and subsequently upregulating the expression of anti-apoptotic proteins. In addition, clinical analyses revealed the presence of the IL-6R/STAT3/miR-204 loop in EOC tissue and showed that reduced miR-204 or elevated IL-6R levels were associated with cDDP resistance and disease progression.

The first component of the circuit linked IL-6 to miR-204 expression via the regulation of STAT3 activation. Exogenous IL-6 stimulation increased STAT3 phosphorylation, and reduced miR-204 expression, suppressed cDDP-induced apoptosis, and STAT3 siRNAs or pharmacologic inhibition abrogated the IL-6-mediated suppression of miR-204 expression. Importantly, restoring miR-204 expression by either miR-204 mimic or STAT3 suppression in IL-6-treated cells restored cDDP sensitivity. The second component of the circuit connected miR-204 to STAT3 activity via the regulation of IL-6R. An inverse correlation between miR-204 and IL-6R expression was detected in EOC cells and tissue. To assess the activity of the feedback loop circuit, we either overexpressed miR-204 or treated cells with IL-6R in combination with miR-204. Elevated miR-204 sensitized EOC cells to cDDP and was accompanied by IL-6R down-regulation and STAT3 inactivation. The knockdown of IL-6R phenocopied the sensitizing effect of miR-204, whereas ectopic IL-6R expression rescued the STAT3 activation and proliferation ability attenuated by miR-204. Moreover, miR-204 effectively suppressed the development of tumor growth in an animal model of EOC, which was impaired by IL-6R re-expression. However, miR-204 did not markedly affect EOC cell growth *in vivo* in the absence of any chemotherapeutic challenge (Supplementary Figure 5C and 5D). This difference in sensitivity may be due to increased levels of detectable apoptosis in miR-204-expressing cells following chemotherapy. In the subsequent mechanistic study, the IL-6-activated STAT3 transcription factor bound directly to the TRPM3/miR-204 gene promoter area, and this effect was enhanced by the presence of IL-6 and reversed by inhibition of STAT3. This binding reduced the levels of miR-204, which impaired the targeting effect of miR-204 to the 3′ UTR of IL-6R to reinforce IL-6R/STAT3 activation. These findings can help tailor the use of chemotherapy in patients with EOC expressing high levels of miR-204 to maximize efficacy and minimize the collateral effects of chemotherapy.

To activate STAT3, IL-6R requires the ligand IL-6 generated by tumor cells or tumor stromal cells, such as macrophages or fibroblasts [42]. According to our data, initial IL-6 stimulation was followed by a perturbation of the other members of the loop, but the loop did not necessarily begin with IL-6 upregulation. This circuit can also be triggered by other extrinsic signals from the tumor microenvironment or intrinsic cancer cell signals (such as oncogene activation and tumor suppressor inactivation), which activate IL-6R or STAT3. Interestingly, miR-204 has been found to be epigenetically silenced by tumor-specific methylation in cancer cells. Therefore miR-204 down-regulation may be the first event that triggers EOC carcinogenesis [29]. Once activated, the circuit is permanently activated due to its positive feed-forward nature, whereas the constitutive activation of the components in the circuit is interdependent: each component regulates its own set of downstream genes that together drive cancer progression. Moreover, several known targets of the components of the IL-6R/STAT3/miR-204 loop might be important for cancer progression. For example, STAT3 can directly induce the expression of the proliferation activator VEGF [43] as well as the chemoresistance inducers IGF-2 and MDR1 [44, 45]. Furthermore, Bcl-2 and TrkB, which are well-established suppressors of apoptosis, are also targets of miR-204 [25]. Together, these data suggest that the initial event that activates this circuit could differ by patient.

Thus, we provide a comprehensive mechanism that contributes to the chemoresistance of EOC: IL-6 activates IL-6R/STAT3 to regulate the expression of miR-204, which controls IL-6R expression. The reversible nature of the IL-6R/STAT3/miR-204 loop permits multiple therapeutic interventions that block its prometastatic activity, and clinical trials for miRNA therapy are currently ongoing [46]. Compared with other strategies, such as siRNA-based therapies, one advantage of miRNA therapy may be its ability to concurrently target multiple genes that are associated with the same network [47]. Although further studies of miRNA delivery, potential off-target effects, and safety are required, our findings suggest that the miR-204-mediated modulation of mechanisms involved in chemoresistance may be a reasonable strategy to maximize the efficacy of conventional chemotherapy for the treatment of EOC.

## MATERIALS AND METHODS

### Cell culture

The human ovarian cancer cell line A2780 and its related cDDP-resistant cell lines (ACRP) were obtained from NANJING KEYGEN BIOTECH CO., LTD, China. SKOV3, OVCAR3, OV2008 and C13* cells, a human breast cancer cell line (MCF-7) and the ADM-resistant cell line (MCF-7/ADM) were obtained from the Shanghai Institute of Cell Biology at the China Academy of Sciences. The culture conditions are described in the Supplementary Methods.

### Tissue samples

With the approval and support of the Jiangsu University ethics committee, serous ovarian cancer samples from patients with FIGO stage IIIC or IV disease (n = 64) were collected at Zhenjiang Maternal and Child Health Hospital (The Fourth Affiliated Hospital of Jiangsu University) and The Affiliated Hospital of Jiangsu University; 10 of these patients experienced recurrence after the first line of platinum-based chemotherapy and had undergone two surgeries at the Zhenjiang. Their tumor material was available to compare the primary and recurrent tumor. All patients were treated with the standard care for platinum-based therapy after surgery, and informed consent was obtained from all patients. PFS was calculated from the time of surgery to the time of progression or recurrence. Platinum resistance or platinum sensitivity was defined as a relapse or progression within 6 months or 6 months after the last platinum-based chemotherapy application, respectively. Clinical and pathological features are described in Supplementary Table 1.

### Reverse transcription quantitative real-time PCR

As previously described [48], the detailed procedure and the sequence of each oligonucleotide are described in the Supplementary Methods. All PCR reactions were performed in triplicate. Changes in gene expression were calculated.

### Transfection

Cells were transfected as previously described [48], and additional details are described in the Supplementary Methods.

### Western blot assay

As described previously [48], proteins (40-50 mg) from cells or tissues were separated by SDS-PAGE and then transferred to polyvinylidene difluoride membranes (PVDF; Bio-Rad, USA). The membranes were blocked and then probed with antibodies against IL-6 (ab6672) or IL-6R (ab128008) (Abcam, Cambridge, MA, USA). p-STAT3 antibody (tyr705) (#4113) was purchased from Cell Signaling Technology, Danvers, MA, USA. The following antibodies were acquired from Santa Cruz Biotechnology (Santa Cruz, CA, USA): Mcl-1 (sc-819), Survivin (sc-8807) and Bcl-2 (sc-7382). Antibodies against Bax (B3428) and β-actin (A9044) were obtained from Sigma (St. Louis, MO). After washing, the blots were incubated with horseradish peroxidase-conjugated secondary antibodies.

### Assessing chemosensitivity to cDDP

Cells were plated in 96-well plates (5×10^3^ cells/well) and exposed to various doses of cDDP (0.01, 0.05, 0.25, 1.25, 7.5 and 25 μM) with or without recombinant human IL-6 ligand (PEPROTECH, NJ, USA). Subsequently, 10 μl of CCK-8 solution was added to each well, and the plate was incubated for 2 h in a humidified incubator. The absorbance of each well was measured at 450 nm using a Model 550 series microplate reader (Bio-Rad Laboratories). Cell viability was expressed as the ratio of treated cells to untreated controls at each dose or concentration. The IC50 value for each cell line was determined with a nonlinear regression analysis using GraphPad Prism (GraphPad Software Inc., San Diego, CA).

### Evaluation of apoptosis

The flow cytometry (FCM) analysis was performed as previously described [48]. After transfection, the cells were treated with cDDP for 24 h, and cell death was evaluated with Annexin-V-FITC and propidium iodide (PI) double staining using a dead cell apoptosis kit according to the manufacturer's protocol (556547, Annexin V-FITC Apoptosis Detection Kit I, BD Biosciences, San Jose, CA, USA). Cells positive for Annexin-V and/or PI were considered dead. Caspase 3 activity was evaluated using the Caspase-Glo®3/7 Assay (G8091, Promega, Madison, Wisconsin, USA), and luminescence was recorded using a Synergy Multi-Mode Plate Reader (Biotek).

### Immunohistochemistry

The tumors were fixed in formalin, embedded in paraffin, sectioned and then heat-immobilized or pepsin-immobilized according to the manufacturer's instructions. The slides were stained with hematoxylin (H&E) or incubated with antibodies against IL-6 (ab6672), IL-6R (ab128008), p-STAT3 (tyr705) (#4113) or Ki-67 (sc-56320) (Santa Cruz Biotechnology, Santa Cruz). Staining was then detected using the Dako Envision two-step method for immunohistochemistry (Carpinteria, CA, USA).

### Immunofluorescence

After treatment, the cells were plated on coverslips, fixed in 4% paraformaldehyde, permeabilized, blocked with 3% BSA, and incubated with p-STAT3 (tyr705) (#4113) for 1 h at 37°C. The cells were then washed and incubated with the secondary antibody for 1 h at room temperature, and confocal images were acquired.

### Chromatin immunoprecipitation (ChIP)-PCR

Chromatin immunoprecipitation (ChIP) assays were performed as previously described [25, 31]. Anti-p-STAT3 antibody (Tyr705) and rabbit isotype IgG were purchased from Cell Signaling Technology (Danvers, MA). STAT3-binding sites surrounding TRPM3 were predicted using an in silico analysis (University of California, Santa Cruz Genome Browser and ENCODE database). Enrichment was detected by quantitative real-time PCR using SYBR green (Takara) and calculated with the comparative Ct method. VEGF was used as a positive control, and IgG was used as a negative control. The primers used are shown in Supplementary Table 2.

### Luciferase reporter assays

Luciferase assays were performed using the Dual-Luciferase Reporter assay system (Promega, Madison, WI, USA) as described previously^48^. The procedures and materials are described in detailed in the Supplementary Methods.

### Tumor xenograft studies

Approximately 5×10^6^ A2780 or SKOV3 cells transfected with mimic-NC containing an empty vector (NC+vector), mimic-NC containing IL-6R recombinant plasmid (NC+IL-6R), miR-204 mimic with an empty vector (mimic+vector), or miR-204 mimic with IL-6R recombinant plasmid (mimic+IL-6R) were intraperitoneally injected into 4-week-old female BALB/c nude mice (SLAC Laboratory, Shanghai, China) (n=6 mice per group). The mice were housed and maintained under specific pathogen-free conditions. When the mice had developed palpable tumors (a week after injection), cDDP (5 mg/kg) was intraperitoneally administered twice per week for 4 consecutive weeks. Because the expression of miRNA diminishes over time, the mice were injected with mimic or mimic-NC (200 μg/kg/mouse, incorporated into DOPC liposomes) twice weekly starting 1 week after tumor inoculation to boost the effectiveness of tumor growth suppression. The tumors were harvested after an additional 4 weeks of cDDP therapy, and the ratio of nodules to mice was calculated.

### Statistical analyses

The data are presented as the mean ± SD from at least three independent experiments. Statistical analyses were performed using an analysis of variance or a two-tailed Student's t-test at the significance level of P< 0.05 (*P<0.05 and **P<0.01). Survival was analyzed based on Kaplan-Meier curves with the log-rank test and a Cox proportional hazard analysis. Spearman's non-parametric correlation test was performed to test the correlation between the expression levels of miR-204 and IL-6R using GraphPad Prism 5 (GraphPad Software, Inc., La Jolla, CA, USA).

## SUPPLEMENTARY FIGURES AND TABLES


