# Supplementary material for: IL-6R/STAT3/miR-204 feedback loop contributes to cisplatin resistance of epithelial ovarian cancer cells
Source: Oncotarget. 2017 Mar 27;8(24):39154–66. doi: 10.18632/oncotarget.16610 (PMC5503602; doi:10.18632/oncotarget.16610)
Supplement: Supplementary file 1 [file oncotarget-08-39154-s001.pdf]

IL-6R/STAT3/miR-204 feedback loop contributes to cisplatin resistance of epithelial ovarian cancer cells

SUPPLEMENTARY MATERIALS

Cell culture

Cells were cultured in DMEM supplemented with 10% fetal calf serum (Gibco BRL, Grand Island, NY), 100 U/mL penicillin and 100 μg/mL streptomycin in a humidified atmosphere containing 5% CO_2 at 37°C.

Transfection

Cells were seeded into 6-well plates (6×10^5 cells/well) and transfected with miR-204 mimic (5'-UUCCCUUUGUCAUCCUAUGCCU-3',5'-GCAUA GGAUGACAAAGGGAAUU-3') or inhibitor (INH,5'-AG GCAUAGGAUGACAAAGGGAA-3') and their negative controls (mimic-NC, 5'-UUCUCCGAACGUGUCAC GUTT-3', 5'-ACGUGACACGUUCGGAGAATT-3' and INH-NC, 5'-CAGUACUUUUGUGUAGUACAA-3', respectively) (all transcripts from Gene Pharma Company, Shanghai, China) at a final concentration of 100 nM using Lipofectamine 2000 (Invitrogen, CA, USA). IL-6R siRNA (siIL-6R, sense: 5'-GCAGGCACUUACUACUAAUTT-3', antisense: 5'- AUUAGUAGUAAGUGCCUGCTT-3'), STAT3 siRNA (siSTAT3, sense: 5'-GGGACCUGGUGU GAAUUAUTT-3', antisense: 5'-AUAAUUCACACCAGG UCCCTT-3'), and negative control siRNA (siNC, 5’-UUCUCCGAACGUGUCACGUTT-3’) were obtained from Qiagen, Inc. (Valencia, CA, USA). The recombinant plasmid eukaryotic expression vector pcDNA3.1-IL-6R (IL-6R) and the empty vector control (vector) were purchased from Cell Signaling Technology, Inc. (Beverly, USA). siIL-6R, siSTAT3, SiCont, IL-6R or vector were transfected into cells using Lipofectamine 2000 according to the manufacturer’s protocol.

Reverse transcription quantitative real-time PCR

Total RNA was extracted from cells or the tissue samples using TRIzol (Invitrogen, Inc., Carlsbad, CA), and cDNA was generated using the PrimeScript® RT Reagent Kit (Takara Co., Ltd., Dalian, China). A quantitative real-time PCR (qRT-PCR) assay was performed to evaluate mature miRNAs and mRNA expression using the SYBR Premix Ex TaqTM II (Takara Co., Ltd., Dalian, China). The relative expression levels of each sample were measured using the $2^{-\Delta\Delta CT}$ method. The primers were as follows: miR-204, forward primer (FW): 5’-GGGCTTCCCTTTGTCATCCT-3’, reverse primer (RV): 5’-GTGCAGGGTCCGAGGT-3’. IL-6R FW: 5'-TTGTTTGTGAGTGGGGTCCT-3', RV: 5'-TGGG ACTCCTGGGAATACTG-3', STAT3: FW: 5'-GGGAAGA ATCACGCCTTCTAC-3', RV: 5'-ATCTGCTGCTTCTC CGTCAC-3'. IL-6: FW: 5'-CAATCTGGATTCAATGA GGAGAC-3', RV: 5'-CTCTGGCTTGTTCCTCACTAC TC-3'. Bcl-2: FW:5'-GGTCATGTGTGTGGAGAGC-3'; RV: 5'-GATCCAGGTGTGCAGGTG-3'. Survivin: FW: 5'-AGCCCTTTCTCAAGGACCAC-3' RV:5'-GCACTTT CTCCGCAGTTTCC-3’. Bax: FW: 5'- GTTTCATCCAG GATCGAGC-3’, RV: 5'-GGAAGTCCAATGTCCAGC-3'. Mcl-1: FW:5'-GTAAGGACAAAACGGGAC-3', RV: 5'-CTCTTCAATCAATGGGGA-3'. β-actin FW: 5'-GCTT CTCCTTAATGTCACGC-3', RV: 5'-CCCACACTGTGCC CATCTAC-3'. The relative levels of miRNA and mRNA were normalized to U6 snRNA and β-actin, respectively.

Luciferase reporter assays

The putative target sites of the human IL-6R 3’UTR segments for miR-204 were amplified using Pyrobest DNA polymerase (Fermentas, CA) and then cloned into the Xba I site of the pGL3 control plasmid (Promega, Madison, USA). The pRL-TK Vectors expressing Renilla luciferase was used as a control vector. miR-204 binding sites in the IL-6R 3’UTR were amplified by PCR with the following primers: IL-6R 3’UTR FW, 5’-GTTTTCCACTGTGGGCTTGT-3’, and RV,5’-TACTGACCCTTTGCCCCATA-3’. The respective mutated putative miR-204 binding site in the IL-6R 3’UTR (Mut-IL-6R 3’UTR) was generated using the QuickChange Site-Directed Mutagenesis Kit (Stratagene, Cedar Creek, USA) according to the manufacturer’s protocol. For reporter assays, HEK293, A2780 and SKOV3 cells were transiently cotransfected with the pRL-TK Vectors (Promega), wild-type or mutant 3’UTR of IL-6R and miR-204 mimic or mimic-NC by Lipofectamine 2000. The firefly and Renilla luciferase activities were measured consecutively using the Dual-Luciferase Reporter assay system (Promega). The luminescence intensity of firefly luciferase was normalized to that of Renilla luciferase.

ELISA assay

This sandwich ELISA assay (cat.no 171-V22552, Bio-rad, USA) assessed the phosphorylation status of tyrosine 705 of STAT3 protein in EOC cells according to instructions. IL-6 levels in EOC cells was evaluated by ELISA assay (R&D Systems, cat. no D6050, USA) following the manufacturer's protocol.

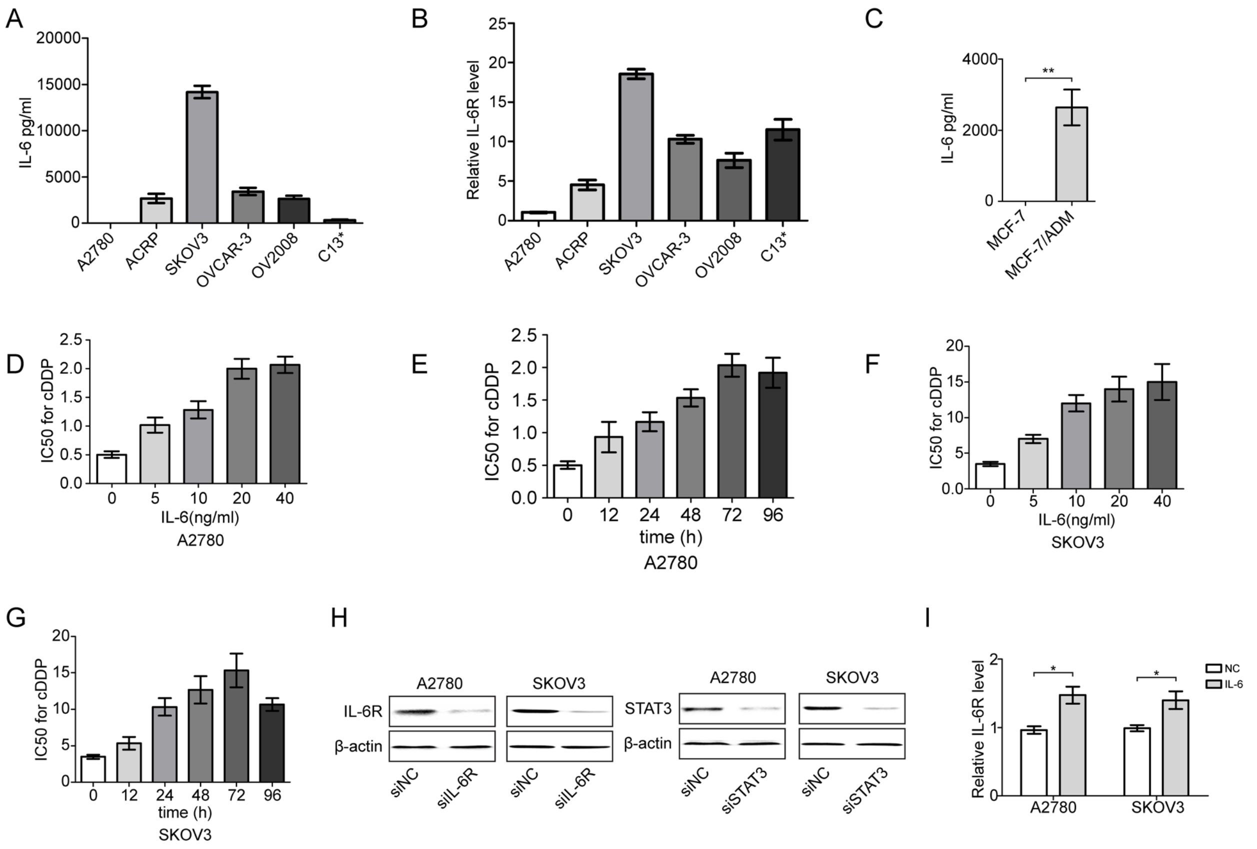

Supplementary Figure 1: IL-6 induces cDDP resistance of EOC cells. **(A)** IL-6 expression level in EOC cells evaluated by ELISA. **(B)** Relative IL-6R mRNA levels in EOC cells. **(C)** IL-6 expression in ADM-sensitive and ADM-resistant breast cancer cell lines. **(D)** IC50 for cDDP in A2780 cells treated with an increasing concentration of IL-6. **(E)** IC50 for cDDP in A2780 cells treated with IL-6 (20 ng/ml) at different time points. **(F)** IC50 for cDDP in SKOV3 cells treated with an increasing concentration of IL-6. **(G)** IC50 for cDDP in SKOV3 cells treated with IL-6 (20 ng/ml) at different time points. **(H)** The protein levels of IL-6R or STAT3. **(I)** Changes in IL-6R mRNA expression in EOC cells after IL-6 treatment. *P< 0.05, **P< 0.01.

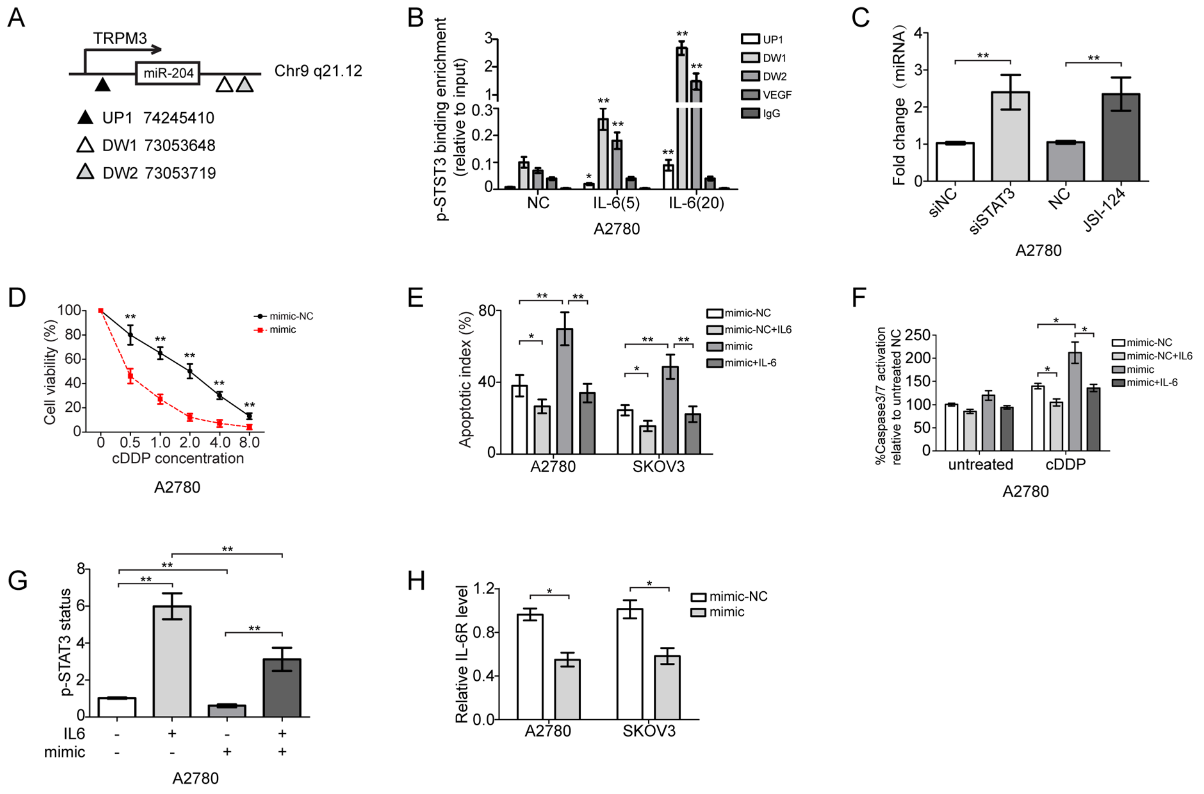

Supplementary Figure 2: IL-6 induces cDDP resistance of EOC cells through direct repression of miR-204 by STAT3. **(A)** Schematic representation of the miR-204 locus and its host gene, TRPM3. STAT3-binding sites around the TRPM3 start site (UP1, DW1 and DW2) were predicted by an in silico analysis. **(B)** ChIP analysis of STAT3 occupancy (fold enrichment) at the TRPM3/miR-204 locus in A2780 cells treated with vehicle or IL-6. **(C)** qRT-PCR analysis of miR-204 in A2780 cells treated with siSTAT3 or JSI-124 (5 µg/ml) for 24 h and subsequently treated with IL-6 for 72 h. EOC cells were treated with mimic for 24 hours and subsequently treated with IL-6 for 72 h, and cell viability **(D)**, cell apoptosis **(E)**, and caspase 3/7 activation **(F)** were measured in EOC cells. **(G)** STAT3 phosphorylation status (Tyr 705) assessed by ELISA in A2780 cells. **(H)** Changes in IL-6R mRNA expression in IL-6-treated EOC cells in response to miR-204 expression. *P< 0.05, **P< 0.01.

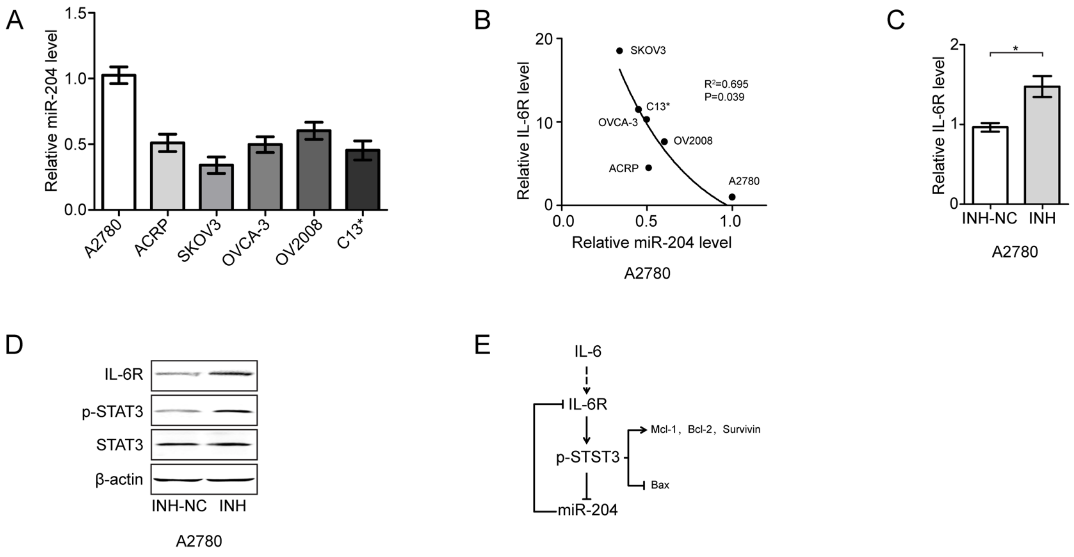

Supplementary Figure 3: miR-204 targeting of IL-6R in EOC cells. **(A)** Relative miR-204 levels in EOC cells. **(B)** miR-204 and IL6R levels in the indicated cell lines and a correlation coefficient (r) are shown. **(C)** Relative IL-6R mRNA in inhibitor-treated A2780 cells. **(D)** The protein levels of IL-6R, p-STAT3 and STAT3 in inhibitor-treated A2780 cells. **(E)** Schematic representation of the proposed IL-6R/STAT3/miR-204 feedback loop. **P*< 0.05, ***P*< 0.01.

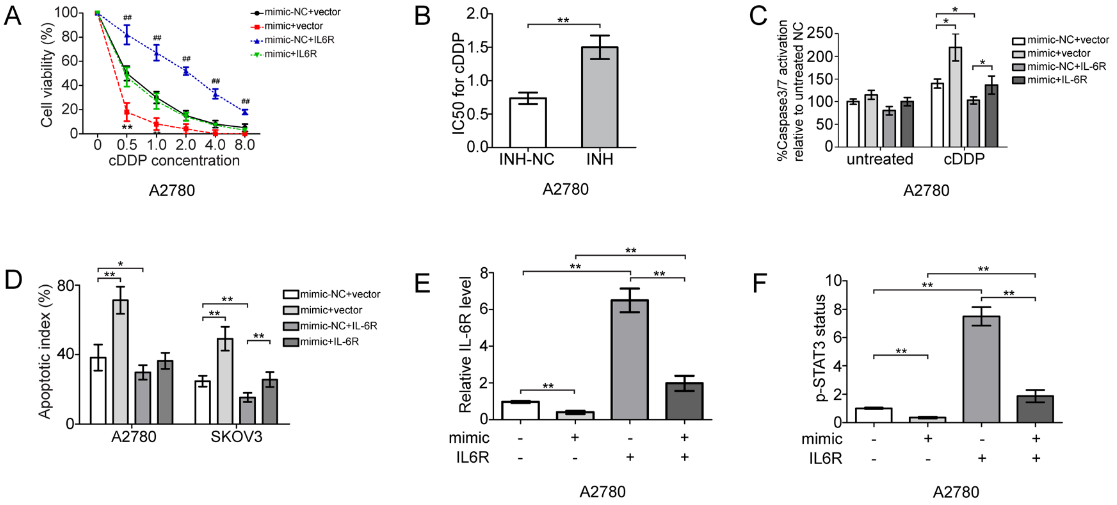

Supplementary Figure 4: Effect of miR-204 on the cDDP sensitivity of EOC cells. A2780 cells were transfected with mimic-NC and empty vector (NC+vector), mimic-NC and IL-6R recombinant plasmid (NC+IL-6R), miR-204 mimic and empty vector (mimic+vector), or miR-204 mimic and IL-6R recombinant plasmid (mimic+IL-6R). After transfection, the change in the cell viability **(A)**, IC50 for cDDP **(B)**, Caspase 3/7 activation **(C)** and cell apoptosis **(D)** were analyzed. **(E)** Changes in IL-6R mRNA expression in A2780 cells. **(F)** STAT3 phosphorylation status (Tyr 705) assessed by ELISA in A2780 cells. *$P < 0.05$, **$P < 0.01$.

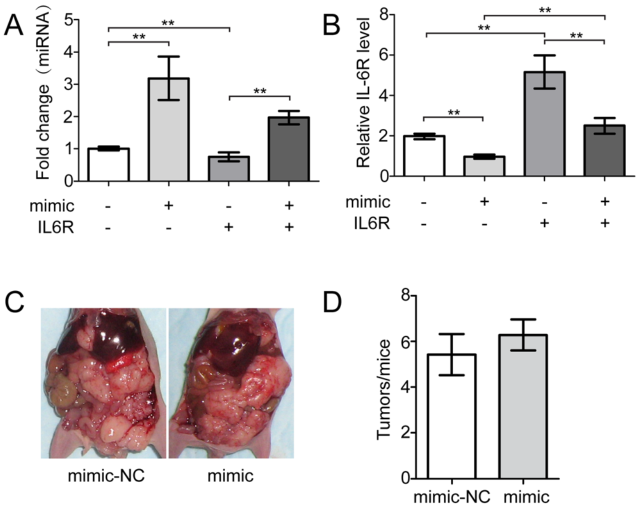

Supplementary Figure 5: Effect of miR-204 on tumor growth *in vivo*. SKOV3 cells transfected with mimic-NC or mimic were intraperitoneally injected into BALB/c nude mice, n=6 per group. **(A)** Relative miR-204 level in SKOV3 xenograft tumors. **(B)** Relative IL-6R mRNA level in SKOV3 xenograft tumors. **(C)** The gross morphology of tumors in nude mice 35 days after tumor cell injection. **(D)** Tumor incidence in indicated mice. *P< 0.05, **P< 0.01.

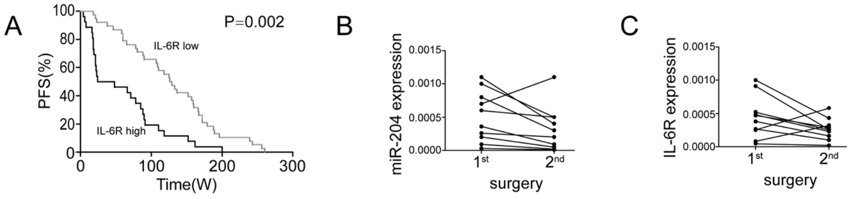

Supplementary Figure 6: (A) A Kaplan-Meier analysis of the PFS of ovarian cancer patients with the corresponding expression profiles of IL-6. Difference in miR-204 **(B)** and IL-6R **(C)** expression between the first (1st) and second (2nd) surgery as quantified in 10 patients.

Supplementary Table 1: Clinicopathological characteristics of OC patients with either high or low miR-204 expression. (N= 64)

		miR-204 low	miR-204 high
Age (yrs)		53.25±7.99	55.01±8.16
Stage	IIIC	26(40.63%)	28 (43.75%)
	IV	6(9.38%)	4(6.25%)
Grade	High	3(4.69%)	2(3.13%)
	Moderate	9(14.06%)	7(10.94%)
	Low	23 (39.94%)	20(31.25%)

Supplementary Table 2: Primers used for quantitative real-time PCR

mRNA	Forward primer	Reverse primer
TRPM3 U1	5'-AACTCATCCCTGGAAGCAAACTGC-3'	5'-TTTGGGCCTCAAGGAAGCAAACTG-3'
TRPM3 D1	5'-ATGTTCCAGGAAGAGGGAACAGCA-3'	5'-TTCTACCCAGAACCTTCCTTCCCA-3'
TRPM3 D2	5'-AAGGAAGTGACTCACAGGAAGGCA-3'	5'-GGCTTGCTGTTGCCCTTGGATAAA-3'9
VEGF	5'-CATACGTGGGCTCCAACAGG-3'9	5'-CGGAGAAGCTGTGTGGTTCCG-3'9
